# Second hand smoke and COPD: lessons from animal studies

**DOI:** 10.3389/fphys.2014.00144

**Published:** 2014-04-10

**Authors:** Michael T. Borchers, Adelheid Kratzer, Laimute Taraseviciene-Stewart

**Affiliations:** ^1^Division of Pulmonary, Critical Care, and Sleep Medicine, Department of Internal Medicine, University of Cincinnati College of MedicineCincinnati, OH, USA; ^2^Center for Molecular Cardiology, University of ZurichSchlieren, Switzerland; ^3^Division of Pulmonary Sciences and Critical Care Medicine, Department of Medicine, School of Medicine, University of ColoradoDenver, CO, USA

**Keywords:** COPD, second hand smoke, mouse models, pulmonary inflammation, emphysema

This issue of Frontiers in Physiology presents a collection of manuscripts including original research and comprehensive reviews that examine the effects of second hand smoke (SHS) on the development and progression of chronic obstructive pulmonary disease (COPD). The health effects and costs of SHS are a burgeoning concern as it is estimated that more than 126 million people in the United States are exposed to SHS (Menzies, 2011). Although there is a decrease of smokers in the US, the economic aspects of SHS exposure is substantial and often occurs not only at the workplace, but also at personal homes (Max et al., 2012). As described in the accompanying manuscripts, SHS has been associated with an increased risk of cardiovascular disease, several cancers, stroke, and type 2 diabetes mellitus. Several studies have shown associations between SHS and sudden infant death syndrome, acute respiratory infections, ear infections, and the development of asthma in infants and children. SHS can also contribute to the development of COPD in non-smokers and exacerbate COPD pathogenesis in smokers. Unfortunately, relatively little is known about the cellular and molecular mechanisms whereby SHS contributes to these diseases including COPD and as a result, insight from animal models can be challenging to translate properly into the human disease. The purpose of this collection is to present the newest data and updated reviews on the role of SHS in the pathogenesis of COPD with a focus on mechanistic studies performed in animal models.

The original research articles presented in this collection examine the influence of dietary vitamin D levels on the susceptibility to SHS-induced pathology, the effects of SHS in a model of pre-existing pulmonary inflammation induced by the genetic deletion of a key leukocyte signaling molecule (Arhgef1), and the role of STAT3 (a master transcriptional regulator of pro-inflammatory genes) in the control of inflammation and protease expression in the lung. In the report by Crane-Godreau et al. (2013), the authors utilized a novel mouse model of dietary vitamin D deficiency and SHS exposure to examine the hypothesis that low vitamin D levels contribute to the lower lung function and disease severity in response to cigarette smoke exposure. These studies revealed that vitamin D deficiency exacerbates the alveolar tissue destruction compared to mice receiving a vitamin D supplemented diet following 16 weeks of daily SHS exposure. Furthermore, the authors provide evidence of a significant shift in the protease-antiprotease balance as a consequence of vitamin D depletion that provides mechanistic insight into the pathways affected by vitamin D deficiency. This work establishes a useful model to further study the effects of dietary vitamin D and the development of pulmonary pathology in response to SHS. The work of Harney et al. also utilizes a novel model to examine the effects of sub-chronic SHS exposure on pulmonary inflammation and tissue destruction (Hartney et al., 2012). Utilizing a mouse genetically deficient in the leukocyte signaling molecule Arhgef, the authors were able to demonstrate that SHS has the capacity to significantly enhance underlying pulmonary inflammation and tissue destruction. Importantly, the data revealed that the effects of the SHS were independent of the baseline inflammation in the mutant mouse model. Therefore, this model is extremely useful for extrapolating and defining the mechanisms of SHS-enhanced pathogenesis in the context of pulmonary inflammation unrelated to smoke exposure. Lastly, Geraghty et al. utilize a mouse model deficient in STAT3 to demonstrate the importance of this critical signaling pathway in the pulmonary response to SHS (Geraghty et al., 2013). The authors showed that tobacco smoke activates STAT3—mediated inflammation, proteolysis, and apoptotic responses in the lung. They further provide a link between the activation of STAT3 and the inhibition of pathways that modulate the anti-inflammatory pathways in the lung.

In addition to the original reports, this issue also presents focused reviews on the effects of SHS on airway secretions and mucociliary clearance (Liu and Di, 2012), the generation and physiological consequences of smoke-induced advanced glycation end-products (Robinson et al., 2012), and the current factors that have been reported in animal models to influence SHS-induced COPD pathologies (Birru and Di, 2012). Finally, the issue presents two comprehensive reviews that detail current understanding of the effects of SHS on lung pathogenesis that has been defined by utilizing animal models, which is a challenging aspect as different rodents develop emphysema at different time points. Goldklang et al. provide a provocative and informative discussion of the lessons learned from animal models of smoke exposure including the strengths and weaknesses of the various models (Goldklang et al., 2013). The Taraseviciene-Stewart lab rounds off the issue with the most current and comprehensive review to date of the effects of tobacco smoke induced pathology described in animal models (Leberl et al., 2013). This review thoroughly summarizes the results of 155 studies addressing cigarette smoke exposure in animal models. Parameters including, species, strain, exposure methodologies, exposure duration and dose, endpoint descriptors, and summaries of the results are thoroughly presented in tables and in discussion formats. In addition to the most common endpoints described in smoke-induced pathologies, this review expands the possibilities and current knowledge of the usefulness of animal studies to investigate small airway remodeling and pulmonary hypertension.

Together, these reports provide insight into the usefulness of animal models of SHS exposure to define potential mechanisms of action in pulmonary pathology as well as timely, thorough updates on our current understanding of the pathologic effects of SHS. Given the tremendous numbers of persons exposed unwillingly to SHS and the likelihood of an increase in COPD in the coming years, it is imperative that the health effects associated with these exposures are carefully and fully examined. The use of animal models for such studies is necessary and, therefore, it is important that the strengths, limitations and relevance to human disease be explored and discussed.

## Conflict of interest statement

The authors declare that the research was conducted in the absence of any commercial or financial relationships that could be construed as a potential conflict of interest.
